# In Skeletally Immature Children Receiving Radiation for Craniofacial Pathology, Is Success of Subsequent Orthopedic Treatment of Maxillary Transverse Skeletal Deficiency Affected by Inclusion of the Midpalatal Suture in Proton Beam Volume?

**DOI:** 10.1016/j.adro.2021.100671

**Published:** 2021-02-12

**Authors:** Chad M. Rasmussen, Nadia N. Laack, Thomas J. Salinas, Olivia M. Muller, Sarah K.Y. Lee, Alan B. Carr

**Affiliations:** a Department of Dental Specialties, Mayo Clinic, Rochester, Minnesota; b Department of Radiology, Mayo Clinic, Rochester, Minnesota

## Introduction

Orthopedic manipulation of the craniofacial skeleton allows for treatment of maxillary growth deformities via circumaxillary suture distraction promoting anterior-posterior protraction or transverse expansion,^1^^,^^2^ or suture compression that restricts normal downward and forward growth.^3^ Proton beam therapy provides a means to include or exclude anatomy from large doses of radiation. This provides distinct advantages over photons as the exit dose may be reduced or eliminated and the exposure field can be customized to avoid sensitive anatomy, thereby providing an opportunity to spare craniofacial sutures necessary for normal growth or correction of growth deficiencies.

A comprehensive literature search at the Mayo Clinic medical library was unsuccessful in finding documented effects of radiation therapy on the fibrous tissue of a craniofacial suture. Known effects of head and neck radiation include hypoplasia of facial bones, facial asymmetry, and dysfunction affecting dental, optical, and auditory structures.^4^ Surgical correction of radiation induced craniofacial deformities is complex due to the atrophic and inelastic changes to skeletal, microvascular, and soft tissues.^5^ Risks to surgically manipulated areas due to suboptimal wound healing capacity also cannot be overemphasized. Even when surgical procedures are indicated, they must often be delayed until complete skeletal maturity of the other facial structures that have not been affected by radiation. Therefore, facial deformities often remain untreated through adolescence.

Two patients treated at Mayo Clinic with proton beam radiation therapy were subsequently evaluated and treated by the orthodontics and dentofacial orthopedics services. Each was originally diagnosed with different craniofacial pathology; however, they received remarkably similar radiation treatments, differing mainly in the anatomy included in the maximum dosage field. In addition, they presented with similar maxillary transverse skeletal deficiencies warranting similar requirements for orthopedic treatment. A comparison of radiation protocols, dental characteristics, and maxillary transverse skeletal relationships is provided in Table 1. Orthopedic expansion of the maxillary midpalatal suture was recommended for both patients and each responded differently to treatment.

**Table 1:** A comparison of radiation protocols, dental relationships, and maxillary transverse relationships for patient 1 and patient 2

	Patient 1	Patient 2
**Radiation protocol**		
Type of radiation	Protons	Protons
Dosage	5580 cGy	5580 cGy
Fractions	31	31
Duration	6 weeks	6 weeks
Number of fields	4	3
Fields	Right lateral, right anterior oblique, left anterior oblique, left posterior oblique	Left anterior oblique, left anterior-superior oblique, left posterior oblique
**Dental relationships**		
Occlusion	Class II molar, class II canine	Class I molar, class II canine
Upper crowding/spacing	7 mm	6 mm
Lower crowding/spacing	6 mm	4 mm
Overjet (horizontal)	4 mm	3 mm
Overbite (vertical)	6 mm (90%)	6 mm (90%)
Upper incisor inclination	Retroclined	Retroclined
Lower incisor inclination	Retroclined	Retroclined
Upper molar inclination	Buccal tip (L)	Buccal tip (R and L)
Lower molar inclination	Lingual tip (R and L)	Lingual tip (R and L)
Crossbites	Primary canines	None
**Skeletal relationship**		
Transverse diagnosis (Andrews Element III)	Deficient, 4 mm	Deficient, 7 mm

*Abbreviations*: L = left; R = right.

## Methods and Materials

Patient 1 is a 10-year-old white male who at 8 years of age began experiencing congestion on the left side of his nose that was not responsive to primary treatment. Nasal endoscopy with biopsy demonstrated pathologic findings consistent with Ewing sarcoma. Patient 2 is a 12-year-old white male who at 10 years of age began experiencing severe headaches and vomiting. Biopsy was positive for poorly differentiated monophasic synovial sarcoma.

### Tumor management

**Fig. 1:**
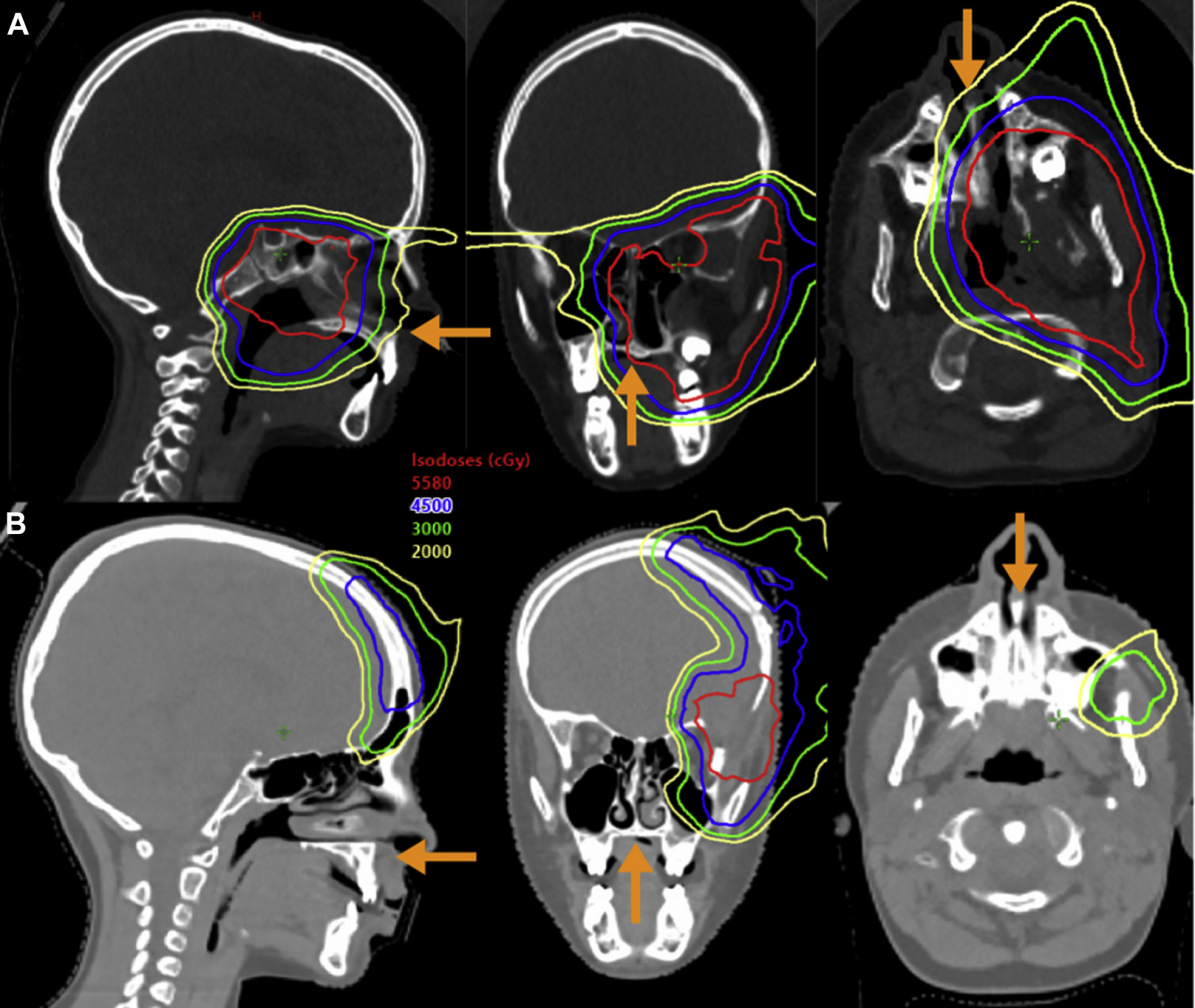
Dosimetry at the maxillary midpalatal suture. The highest dosage (55.8 Gy) area is indicated by the red line. The orange arrow represents the location of the maxillary midpalatal suture in each slice. Isodose line values (cGy): red = 5580, blue = 4500, green = 3000, yellow = 2000. (A) Patient 1. The sagittal and axial slices show the high-dose area includes the posterior aspect of the midpalatal suture region, with dosage declining rapidly as it moves anteriorly. The coronal and axial slices show the medial aspect of the high-dose area ending directly at the midpalatal suture. (B) Patient 2. The highest dosage of radiation was delivered superior and lateral to the midpalatal suture. (A color version of this figure is available at https://doi.org/10.1016/j.adro.2021.100671.)

Both patients received a maximum radiation dose of 55.8 Gy, delivered with pencil-beam proton radiation therapy in 31 fractions over 6 weeks. Isodose curves obtained at the level of the maxillary midpalatal suture are provided for patient 1 (Fig 1A) and patient 2 (Fig 1B). No attempt was made prospectively to spare the suture line. Radiation target volumes were defined as per standard Children’s Oncology Group Ewing sarcoma and soft-tissue sarcoma protocol guidelines. Patient 1 was treated with a 4-field plan using a right lateral, right and left anterior oblique, and left posterior oblique fields. The posterior quarter of patient 1’s midpalatal suture was included in the 55.8 Gy level of radiation. The dosage delivered dropped rapidly toward the anterior aspect of the suture from 45.0 to 30.0 and finally 20.0 Gy. Patient 2 was treated with 3 fields: left anterior oblique, left anterior-superior oblique, and left posterior oblique. Owing to the superficial nature of the tumor, a 4-cm water-equivalent-thickness bolus helmet was used to sharpen lateral penumbra as well as reduce proton energy and allow full dose at the surface. In addition, the superficial location of patient 2’s tumor resulted in sparing of the midpalatal suture from radiation during treatment. Chemotherapy was administered 3 months postradiation for both patients. Patient 1’s regimen consisted of ifosfamide and etoposide given over 5 days, whereas patient 2 received ifosfamide delivered over 3 days.

### Craniofacial deficiency management

**Fig. 2:**
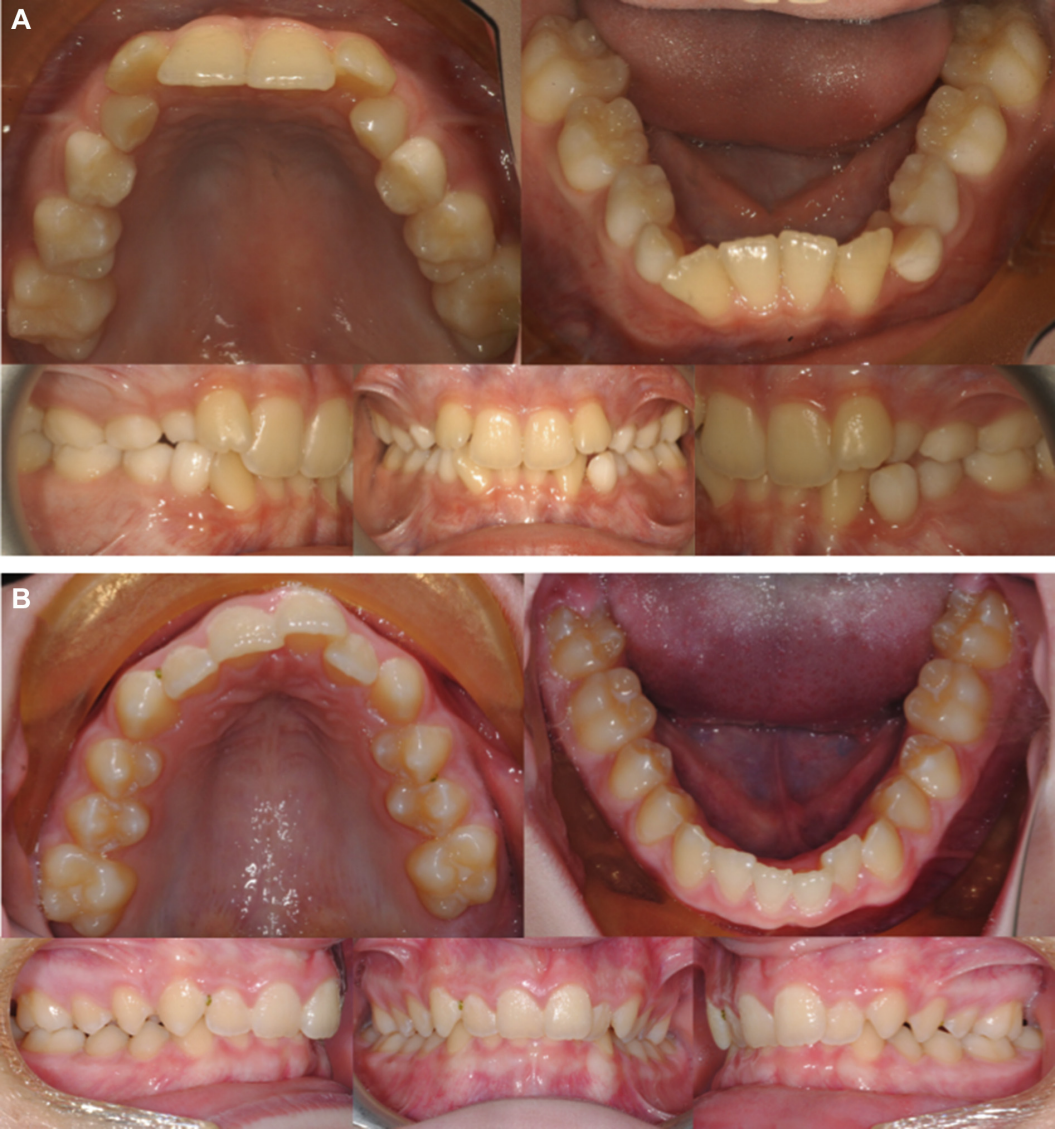
Pretreatment dental relationships. Both patients exhibit maxillary dental crowding, lingually inclined maxillary incisors, and lingually inclined mandibular dentitions. Expansion of the maxillary midpalatal suture is indicated to relieve maxillary crowding and to allow placing the maxillary incisors and mandibular teeth at their proper inclinations. (A) Patient 1, (B) patient 2.

Patient 1 (Fig 2A) had a 4-mm maxillary transverse deficiency compared with a 7-mm deficiency for patient 2 (Fig 2B). Diagnoses were made with Andrews’ Element III method^6^ incorporating both molar inclination and intermolar width. Orthopedic treatment began 12 months after completion of radiation therapy for patient 1 and after 23 months for patient 2. Both patients received a Hyrax-type maxillary expansion appliance with palatal miniscrew (Allesee Orthodontic Appliances, Sturtevant, WI), which was bonded to the maxillary permanent first molars with Bandlok orthodontic cement (Reliance Orthodontic Products, Itasca, IL). With each turn of the expansion screw, the appliance is activated in 0.25-mm increments. The regular appliance activation protocol is 1 turn per day. A conservative approach was used with patient 1, whose midpalatal suture was included in the high-dose area of his radiation map. The activation schedule was decreased to 1 turn every 3 days to ensure adequate vascular perfusion of the suture between force applications. As dosage maps for patient 2 showed no involvement of the midpalatal suture, the regular appliance activation protocol was used.

## Results

**Fig. 3:**
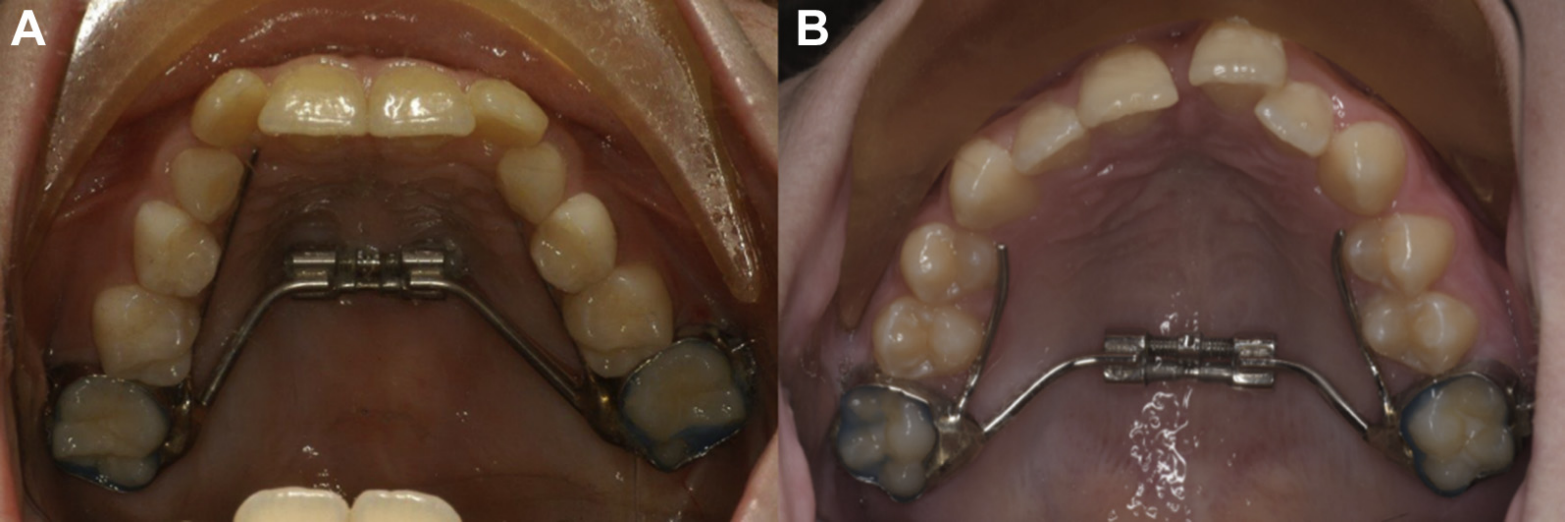
Maxillary dental arches after attempts to orthopedically expand the midpalatal suture. (A) Patient 1. Separation of the expansion screw is shown without change in the maxillary archform or creation of a midline diastema. (B) Patient 2. Separation of the expansion screw with change in the maxillary archform and creation of a midline diastema indicate successful suture expansion.

Patient 1 was recalled for evaluation at 8 weeks (Fig 3A). Palatal tissues appeared well perfused with no signs of negative effects from the appliance or from expansion. The expander was opened 2 mm on either side of the screw; however, no maxillary diastema was present between the central incisors and no change in maxillary archform was seen, indicating unsuccessful expansion of the midpalatal suture. Patient 2 was recalled at 5 weeks (Fig 3B) and demonstrated positive changes to the maxillary archform as well as the presence of a midline interdental diastema, indicating successful orthopedic expansion of the maxillary midpalatal suture.

Both patients have remained disease free since radiation and chemotherapy treatment. Patient 1 received magnetic resonance imaging examination 25 months after treatment with no signs of recurrent pathology. Because orthopedic expansion was unsuccessful, further orthodontic treatment was delayed until all primary teeth exfoliated. Patient 2 had magnetic resonance imaging accomplished 18 months posttreatment and did not show any evidence of disease. Orthodontic treatment with aligner therapy was initiated and has progressed in a routine manner with no signs of breakdown in the midpalatal suture through 10 months.

## Discussion

Proton beam therapy has become the treatment of choice in high-risk regions of the body where inadvertent exposure of adjacent anatomy can cause serious complications.^7^ In the paranasal region adjacent to the maxillary midpalatal suture, a meta-analysis by Patel et al^8^ demonstrated how charged particle therapy is especially advantageous over photon therapy. An anatomy-exclusive radiation protocol is particularly salient in pediatric head and neck cancers to limit long-term adverse effects to physical and cognitive function, development of secondary cancers,^9^ and development of craniofacial hypoplasia and asymmetry.^10^ Preliminary data from Vern-Gross et al^11^ suggest the sharp dosimetry gradient associated with proton beam therapy is not associated with an increased risk of marginal failure, which is a positive report for this proposed protocol. However, sparing growth sutures should only be considered when a patient’s oncologic needs allow for their exclusion. Tumor type, location, and size must take precedence in determining the anatomy to be included in a radiation field.

Orthopedic expansion of the maxillary midpalatal suture in 10- and 12-year-old nonirradiated male patients is expected to be successful.^12^ Craniofacial skeleton growth deformities in children may be caused by genetically mediated growth patterns or acquired through radiation treatment to the head and neck. Although neither of the patients included in this case report were examined for craniofacial growth deficiencies before initiation of radiation therapy, the relatively short duration between radiation and diagnosis of skeletal deficiency suggests the growth attenuation of these patients is likely secondary to their genetic growth predispositions.

Genetic growth deficiencies develop over the entire adolescent growth period.^13^ Moss and Salentjin^14^ describe growth of the maxilla as dependent on downward and forward pull of the facial soft tissues, which distracts the circumaxillary sutures. This results in deposition of bone at the suture and an increase in bone size. Additionally, expansion of the spheno-occipital and sphenoethmoidal sutures of the cranial base occurs and are independently mediated growth sites pushing the maxilla from behind, also in a downward and forward direction.

Further study needs to be done to determine whether skeletal hypoplasia seen in postradiation children is due to inelasticity of the soft tissue preventing translation of the facial skeleton or to cellular changes within the suture itself. The clinical result of this small sample observation suggests there may be significant calcification or fibrosis of the postradiation suture preventing expansion with the traditional amount of orthopedic force. However, this requires in-depth research with a statistically significant patient population in order to draw definitive conclusions. Such work will need to be done before alterations in radiation planning are considered. Although palatal expansion largely involves midpalatal suture dynamics in the horizontal plane, study into anterior-posterior growth and treatment potential needs to be made.

Beyond the simple inclusion or exclusion of craniofacial sutures, however, this report identifies 2 critical areas where additional investigation is necessary. In 2013, Dörr et al^15^ demonstrated a 20 Gy dose-response threshold above which radiation therapy is associated with growth deformities in noncraniofacial bones. Beyond 35 to 40 Gy, additional doses of radiation did not cause additional growth deformities. A high-quality dose-response threshold for craniofacial sutural growth has yet to be determined, without which it is difficult to determine the benefits of reduction in the low-dose bath afforded by proton treatment in these cases. Based on noncraniofacial bone data, attempting to keep suture line to <35 Gy, ideally <20 Gy, may allow for further bone growth. Indeed, this observation may hold true whether protons or photon treatment is selected, depending on the ability of the radiation oncologist to plan dosimetry that spares the suture. Because sarcomas are generally infiltrative, no attempt was made to limit clinical target volume expansions that crossed the suture line. For tumors with pushing borders or limited evidence of infiltrative disease, it may be reasonable to limit clinical target volume expansion to <1 cm to respect a suture line similar to what is currently accepted practice in long-bones at growth plates in pediatric sarcomas. Future studies with careful follow-up to ensure maintenance of tumor control outcomes are required to evaluate the safety of this practice.

Additionally, the temporal relationship between initiation of radiation therapy and the onset of orthopedic expansion treatment should be evaluated. The question posed is whether initiation of orthopedic treatment for patient 1 sooner than 12 months would have affected his clinical outcome. Taken together, identification of a dose-response curve for sutural growth, a viable time-frame for orthopedic expansion, and the observation of this case report that high-dose radiation of craniofacial sutures affects orthopedic expansion, may suggest early inclusion of dentofacial orthopedic specialists for skeletally immature children undergoing treatment for head and neck cancer.

## Conclusions

This 2-case report indicates that the ability to orthopedically treat maxillary growth deficiency through expansion of a craniofacial suture is dependent upon inclusion of the suture in the high-dose distribution plan. This finding may provide a novel way of evaluating craniofacial growth in skeletally immature children receiving radiation for head and neck cancer, suggest directions for future investigation, and support the development of an anatomy-exclusive radiation protocol that spares craniofacial sutures when able.
